# Resistance to cosmetic botulinum toxin A: A 15-patient case series across 12 sites

**DOI:** 10.1016/j.jdin.2024.08.021

**Published:** 2024-09-28

**Authors:** Carlos G. Wambier, Fatima N. Mirza, Sarah P.F. Wambier, Vince Bertucci, Jean Carruthers, Joely Kaufman, John Martin, J. Barton Sterling, Flávia Brasileiro, Carolina Marçon, Allison J. Brown, Miranda Rosenberg, Lilia R.S. Guadanhim, Doris Hexsel

**Affiliations:** a Department of Dermatology, The Warren Alpert Medical School of Brown University, Providence, Rhode Island; b Division of Dermatology, University of Toronto, Toronto, Canada; c Private Practice, Woodbridge, Canada; d Dr. Jean Carruthers Cosmetic, Vancouver, Canada; e Department of Ophthalmology, The University of British Columbia, Vancouver, Canada; f Skin Associates of South Florida, Coral Gables, Florida; g Private Practice, Coral Gables, Florida; h Jersey Shore University Medical Center, Spring Lake, New Jersey; i Clínica Renascence, Presidente Prudente, Brazil; j Private Practice, São Paulo, Brazil; k Dermatology and Surgery of Southern Ohio, Fairfield, Ohio; l Palm Beach Dermatology, Palm Beach, Florida; m Department of Dermatology, Universidade Federal de São Paulo, São Paulo, Brazil; n Hexsel Dermatology Clinics, Rio de Janeiro and Porto Alegre, Brazil

**Keywords:** botox, botulinum toxin A, botulinum toxin B, cosmetic dermatology, neuromodulator, neurotoxin, rhytids, wrinkles

*To the Editor:* With the increased popularity of cosmetic botulinum neurotoxin type A (BNTA) injections, evidence is emerging on the development of resistance in some patients.^1^ Although the exact prevalence or incidence of this phenomenon is likely modest overall, this study aimed to investigate clinical characteristics of complete BNTA resistance and to compare the effects of alternative neurotoxins attempts after diagnosis.

The authors compiled a dataset stripped of identifiers (Supplementary Dataset, available via Mendeley at https://data.mendeley.com/datasets/m9wcp88h2j/2). Our series included 15 patients with a median onset age of resistance of 46 years (range: 31-76 years) and a median latency for developing resistance of 3 years (range: 0-14 years). Most patients were female (66.6%) and lived in urban areas (86.6%). The most commonly used initial BNTA was OnabotulinumtoxinA (73.3%). The median age at first injection was 40 years (range: 30-76 years). Before resistance, 8 (53.3%) received touch-ups. After resistance developed, the median follow-up duration was 5 years (range: 0.5-20 years). One-third of patients were on their usual dose at the last effective treatment and 26.7% were on escalating dose because of reduction of effect/duration (Table I).

**Table I:** Characteristics of 15 patients with complete resistance to cosmetic botulinum neurotoxin type A

Complete resistance	**Overall (*N* = 15)**		**BNTB (*N* = 7)**		**BNTA (*N* = 8)**	
Median age, first injection, y (range)	40 (30-76)		38 (30-75)		42.5 (30-70)	
Median year, first injection (range)	2012 (1998-2023)		2011 (1998-2015)		2013 (2003-2023)	
Female, *n* (%)	10 (66.6%)		5 (71.4%)		5 (62.5%)	
Rural area, *n* (%)	2 (13.3%)		1 (14.3%)		1 (12.5%)	
First botulinum toxin A injection, *n* (%)						
OnabotulinumtoxinA	11 (73.3%)		5 (71.4%)		6 (75.0%)	
AbobotulinumtoxinA	1 (6.7%)		1 (14.3%)		0 (0.0%)	
IncobotulinumtoxinA	1 (6.7%)		1 (14.3%)		0 (0.0%)	
Unknown	2 (13.3%)		0 (0.0%)		2 (25.0%)	
Other toxins used before onset, *n* (%)						
AbobotulinumtoxinA	2 (13.3%)		2 (28.6%)		0 (0.0%)	
Touch-ups before onset, *n* (%)	8 (53.3%)		3 (42.9%)		5 (62.5%)	
Median age of onset resistance, y (range)	46 (31-76)		44 (31-36)		46 (38-73)	
Median years of resistance follow-up (range)	5.0 (0.5-20)		6 (1-20)		4 (0.5-10)	
Median years of latency to onset (range)	3.0 (0-14)		5 (0-11)		2 (0.5-8)	
Dose of last treatment with effect, *n* (%)						
Usual dose	5 (33.3%)		1 (14.3%)		4 (50.0%)	
Alternate/higher dose	4 (26.7%)		3 (42.9%)		1 (12.5%)	
Alternative neurotoxins attempted after onset, *n* (%)		Response (%)		Response (%)		Response (%)
AbobotulinumtoxinA	11 (73.3%)	1 (9.1%)	6 (85.7%)	0	5 (62.5%)	1 (12.5%)
IncobotulinumtoxinA	7 (46.7%)	1 (14.3%)	4 (57.1%)	0	3 (37.5%)	1 (12.5%)
PrabotulinumtoxinA	3 (20.0%)	0 (0%)	2 (28.6%)	0	2 (25.0%)	0
OnabotulinumtoxinA	2 (13.3%)	0 (0%)	1 (14.3%)	0	1 (12.5%)	0
DaxibotulinumtoxinA	2 (13.3%)	0 (0%)	1 (14.3%)	0	1 (12.5%)	0
Unknown toxin A	1 (6.7%)	0 (0%)	0 (0%)	-	1 (12.5%)	0
RimabotulinumtoxinB	7 (46.7%)	6 (85.7%)	7 (100%)	6 (85.7%)	-	-

Patients are also subdivided to those who trialed toxin B (BNTB = 7) or toxin A only (BNTA = 8). Two out of 15 patients responded to another botulinum toxin A: 13.3%. Six out of 7 patients responded to botulinum toxin B: 85.7%. (*P* = .0023, Fisher’s exact test).

*BNTA*, Botulinum neurotoxin type A; *BNTB*, botulinum neurotoxin type B.

After resistance diagnosis, alternative BNTA treatments were attempted. Among those patients only exposed to alternative BNTA, 2 patients (13.3%) overcame resistance. Out of 7 patients transitioned to botulinum neurotoxin type B (RimabotulinumtoxinB), 85.7% responded; this was significantly higher than the response rate to another BNTA, *P* = .0023 (Fisher’s exact test).

Among RimabotulinumtoxinB responders, resistance was observed in 2 cases, 1 after receiving 3 treatments of 5000 U, and 1 after the second dose of 2500 U, which resolved after a 1-year washout. Five (83.3%) are still receiving RimabotulinumtoxinB with good effect. Overall, the median response duration was 5 weeks (IQR: 1.5-5) and median dose was 5000 U (range: 2500–10,000 U). A dose of 10,000 U had 7-week duration (Table II).

**Table II:** Outcomes of 7 patients who were transitioned to botulinum neurotoxin type B (RimabotulinumtoxinB) after the diagnosis of complete resistance to cosmetic botulinum neurotoxin type A

	(*N* = 7)
RimabotulinumtoxinB (total)	
No response (primary resistance)	1 (14.3%)∗
Response to RimabotulinumtoxinB, *n* (%)	6 (85.7%)
Currently responding	5 (83.3%)
Secondary complete resistance	1 (16.6%)
Secondary complete temporary/partial	1 (16.6%)
Median weeks of duration (IQR)†	5 (1.5-5)
Range, wk	0-7
Average dosage x^-^ (SD)†	4737 U (1645)
Median dosage (IQR)	5000 U (5000-5000)
Dosage range	2500-10,000 U
2500 U RimabotulinumtoxinB (*n*)	4
Median weeks of duration (IQR)	1.5 (1.1-1.8)
Range, wk	0-2.5
5000 U RimabotulinumtoxinB (*n*)	14
Median weeks of duration (IQR)	5 (5-5)
Range, wk	0-6
10,000 U RimabotulinumtoxinB (*n*)	1
Median weeks of duration (IQR)	7 (7-7)
Range, wk	7-7
Median years RimabotulinumtoxinB follow-up (IQR)	3.0 (0.6-5.0)
Range	0.5-6

∗ The patient with primary complete resistance to RimabotulinumtoxinB also had primary resistance to botulinum toxin A.

† Average response duration and dosage calculated for all treatments, including multiple treatments of single patients. Failures were coded as duration = 0 weeks.

This extremely rare resistance to cosmetic BNTA (only 15 cases identified our 12 sites), has been previously attributed to the development of neutralizing antibodies after previous injections^2^ or even more rare primary resistance, which may be caused by pre-existing botulinum neurotoxin type-neutralizing antibody from unnoticed botulism or mutations in receptors that modifying the toxin’s interaction sites.^3^ The effective response to RimabotulinumtoxinB is typically shorter than BNTA responses. In one study, 2000 to 1000 U RimabotulinumtoxinB lasted 11 to 7 weeks, respectively.^4^ For cervical dystonia, 10,000 U RimabotulinumtoxinB lasted 12 to 16 weeks.^5^

Although transitioning to RimabotulinumtoxinB may be considered for selected patients, the management of complete BNTA resistance still requires more clinical studies, particularly on the long-term duration of the response to RimabotulinumtoxinB, longer wash-outs from BNTA, immune suppressive agents, or other botulinum toxin serotypes.

## Conflicts of interest

Dr C. G. Wambier has served a consultant for Allergan Aesthetics, AbbVie Company and as a speaker of Galderma Laboratories and cosmetic dermatologic surgery editor of the Surgical & Cosmetic Dermatology Journal and Brazilian Society of Dermatology (SBD). Dr Bertucci has served as a speaker, consultant, and/or investigator for Allergan Aesthetics, AbbVie Company, Clarion Medical Technologies, Cutera, Evolus, Galderma, L’Oreal, Revance, and Teoxane. Dr Carruthers has served as consultant for Acorn, Alastin, Appiell, Allergan Aesthetics, AbbVie Company, Avari, Bonti (now with Allergan/AbbVie), Del Nova, Evolus, Fount Bio, In Mode, Inverse Genomic Ltd, Jeune Aesthetics, Merz, Revance Biopharma, Object Pharma, Sofwave, is an author and editor for Elsevier, is an author for “Up to Date” Neuromodulators and Fillers, is an assistant editor for Dermatologic Surgery, is a reviewer for the Aesthetic Surgery Journal, Ophthalmic Plastic and Reconstructive Surgery, and Plastic and Reconstructive Surgery. Dr Kaufman has been an investigator for Allergan Aesthetics, AbbVie Company, Croma, Galderma, MedyTox, Merz, and Revance. Dr Guadanhim is speaker and investigator for Allergan Aesthetics and AbbVie Company. Dr Hexsel is a consultant for Galderma Laboratories, and cosmetic dermatology editor of the Surgical & Cosmetic Dermatology Journal and Brazilian Society of Dermatology (SBD). Drs Mirza, S. P. F. Wambier, Martin, Sterling, Brasileiro, Marçon, Brown, and Rosenberg have no conflicts of interest to declare.

## References

[1] Ho W.W.S., Albrecht P., Calderon P.E. (2022). Emerging trends in botulinum neurotoxin A resistance: an international multidisciplinary review and consensus. Plast Reconstr Surg Glob Open.

[2] Torres S., Hamilton M., Sanches E., Starovatova P., Gubanova E., Reshetnikova T. (2014). Neutralizing antibodies to botulinum neurotoxin type A in aesthetic medicine: five case reports. Clin Cosmet Investig Dermatol.

[3] Pirazzini M., Carle S., Barth H., Rossetto O., Montecucco C. (2018). Primary resistance of human patients to botulinum neurotoxins A and B. Ann Clin Transl Neurol.

[4] Lowe N.J., Yamauchi P.S., Lask G.P., Patnaik R., Moore D. (2002). Botulinum toxins types A and B for brow furrows: preliminary experiences with type B toxin dosing. J Cosmet Laser Ther.

[5] Brin M.F., Lew M.F., Adler C.H. (1999). Safety and efficacy of NeuroBloc (botulinum toxin type B) in type A-resistant cervical dystonia. Neurology.

